# Response to Choi et al, “Pathergy beyond neutrophilic dermatoses: A case of localized leukemia cutis at the site of intravenous infiltration”

**DOI:** 10.1016/j.jdcr.2025.11.030

**Published:** 2025-12-01

**Authors:** Sarah J. Hayek, Christopher N. Nguyen, Dario Kivelevitch

**Affiliations:** a School of Medicine, University of Mississippi Medical Center, Jackson, Mississippi; b Department of Dermatology, Baylor University Medical Center, Dallas, Texas

**Keywords:** isotopic response, Koebner phenomenon, neutrophilic dermatosis, pathergy

To the Editor:

We commend Choi et al^1^ for their insightful report, “Pathergy beyond neutrophilic dermatoses: A case of localized leukemia cutis at the site of intravenous infiltration.” The case adds valuable perspective to the spectrum of leukemia cutis presentations. However, we believe the term *pathergy* is inappropriately applied in this context.

Pathergy, by definition, refers to a sterile, nonspecific hypersensitivity reaction to minor trauma, most commonly associated with neutrophilic dermatoses such as pyoderma gangrenosum and Sweet syndrome.^2^ Histopathologically, pathergy demonstrates a neutrophil-predominant infiltrate without true vasculitic features.^3^ The presence of methicillin-susceptible *Staphylococcus aureus* and biopsy findings of atypical cells expressing CD33, CD117, and myeloperoxidase (consistent with leukemia cutis) argue against a pathergic process. The lesion described instead reflects leukemic infiltration arising at an infected, traumatized site.

The authors draw an analogy between leukemia cutis developing at a site of trauma and a pathergic response. However, these processes are distinct, and the terminology should reflect this distinction. This presentation may represent a Koebner phenomenon, in which new lesions of an existing skin disease appear at sites of trauma, or more likely an isotopic response, where a new condition (not necessarily a primary dermatosis) develops in previously traumatized skin.^4^^,^^5^ Each term—pathergy, Koebnerization, isotopic response—carries distinct definitions that should not be used interchangeably. Precise terminology remains essential, as dermatologic descriptors convey specific pathogenetic and diagnostic information that guide clinical interpretation.

## Conflicts of interest

None disclosed.

## References

[1] Choi J., Lei D., Choi S. (2025). Pathergy beyond neutrophilic dermatoses: a case of localized leukemia cutis at the site of intravenous infiltration. JAAD Case Rep.

[2] Ergun T. (2021). Pathergy phenomenon. Front Med (Lausanne).

[3] Ergun T., Gürbüz O., Harvell J., Jorizzo J., White W. (1998). The histopathology of pathergy: a chronologic study of skin hyperreactivity in Behçet’s disease. Int J Dermatol.

[4] Camargo C.M., Brotas A.M., Ramos-e-Silva M., Carneiro S. (2013). Isomorphic phenomenon of Koebner: facts and controversies. Clin Dermatol.

[5] Weiss G., Shemer A., Trau H. (2002). The Koebner phenomenon: review of the literature. J Eur Acad Dermatol Venereol.

